# Results and lessons learned from a prevention of weight gain program for low-income overweight and obese young mothers: *Mothers In Motion*

**DOI:** 10.1186/s12889-017-4109-y

**Published:** 2017-02-10

**Authors:** Mei-Wei Chang, Roger Brown, Susan Nitzke

**Affiliations:** 1 College of Nursing, The Ohio State University, 342 Newton Hall, 1585 Neil Avenue, Columbus, OH 43210 USA; 2 School of Nursing, University of Wisconsin-Madison, 600 Highland Avenue, Madison, WI 53792 USA; 3 Department of Nutritional Sciences, University of Wisconsin-Madison, 1415 Linden Drive, Madison, WI 53706 USA

**Keywords:** Obesity, Intervention, Diet, Stress, Physical activity, Low-income women

## Abstract

**Background:**

*Mothers In Motion (MIM)*, a community-based lifestyle behavioral intervention, was designed and conducted to help low-income overweight and obese young mothers prevent further weight gain via promotion of stress management, healthy eating, and physical activity. This paper presents intervention effect on body weight (primary outcome) and summarizes lessons learned.

**Methods:**

Participants (*N* = 612) were recruited from 7 Special Supplemental Nutrition Program for Women, Infants, and Children (WIC) offices in Michigan and were individually randomized to an intervention *n*= 410) or a comparison (*n* =202) group (2: 1 ratio). During the 16-week intervention, intervention participants watched theory-based culturally sensitive videos (in DVD format) featuring peers from the target audience to learn skills for managing stress, eating healthier, and being more physically active. They also dialed into peer support group teleconferences to enhance skills learned in the videos and increase motivation for lifestyle behavioral changes. Body weight, the primary outcome, was measured at baseline, immediately after the 16-week intervention, and 3 months after the 16-week intervention. Intervention effect was tested via general linear mixed model for repeated measures, using baseline measures as adjusting covariates.

**Results:**

At baseline, the mean age of the participants was 28.5 ± 5.0 years (intervention: 28.4 ± 5.0, comparison: 28.9 ± 5.0); the mean body weight was 190.2 ± 1.4 lbs (intervention: 191.8 ± 30.0, comparison: 188.5 ± 29.1); and the mean body mass index (BMI) was 32.2 ± 4.4 (intervention: 32.2 ± 4.4, comparison: 31.7 ± 4.2). Of sample, 64.7% were obese. At 3 months after the 16-week intervention, no significant weight differences were found between the intervention (188.3 ± 10.6 lbs, BMI: 31.6 ± 1.8) and comparison groups (187.7 ± 10.6 lbs, BMI: 31.53 ± 1.8) when controlling for baseline body weight.

**Conclusions:**

This lifestyle behavioral intervention that focused on stress management, healthy eating and physical activity was not effective in helping low-income overweight and obese young mothers prevent further weight gain.

**Trial registration:**

Clinical Trials NCT01839708. This trial was registered retrospectively on February 28, 2013.

## Background

Obesity is disproportionally high among low-income women in the US [1, 2]. An excellent setting to combat the high prevalence of obesity in this population is the Special Supplemental Nutrition Program for Women, Infants, and Children (WIC). WIC is a federally funded community program that provides food vouchers and nutrition consultation and makes referrals to other services. To be qualified to participate in WIC, individuals must have annual household incomes at or below 185% of the federal poverty line. There have been efforts of WIC nationwide to reduce the prevalence of obesity in child-bearing aged women and young children [3]. When successful, WIC can have a significant impact on public health because WIC serves nearly 10 million clients annually: pregnant, postpartum, and breastfeeding women; infants and children up to age 5 [4]. About half of American children participate in WIC. About 50% of low-income pregnant women enrolled in WIC reported being overweight or obese before becoming pregnant [5]. However, WIC dietitians and nurses face competing demands as they care for a diverse clientele with limited program resources. Therefore, an intervention program that is designed to help WIC clients eat healthier and be more physically active and can be adopted, implemented, and sustained by WIC nationwide to supplement WIC daily practice is critically important to reduce obesity epidemic in the US.

Overweight and obese women are at high risk of excessive gestational weight gain (defined as gaining more weight during pregnancy than the 2009 Institute of Medicine pregnancy weight gain guidelines) [6]. Excessive gestational weight gain is associated with postpartum weight retention [7–11]. Being low-income adds to women’s risk of retaining major weight gain (defined as retaining at least 10 lbs at 1 year postpartum) in the period following pregnancy. At 1 year postpartum, 68% of low-income women experience significant postpartum weight retention, versus 32% of higher-income women [12]. Gaining 3 or more body mass index (BMI) units between pregnancies increases women’s risk for adverse maternal (e.g., gestational diabetes and pre-eclampsia) and birth outcomes (e.g., macrosomia) [13].

Up to date, four pilot lifestyle behavioral intervention studies have been conducted to help low-income overweight and obese young women lose weight [14–17] and possibly reduce their risk for adverse maternal and birth outcomes during the next pregnancy. Results of these 4 studies were disappointing because only 1 study showed that the intervention group lost weight immediately after the 14-week intervention relative to a control group [16]. It is unclear what contributed to the apparent success of this prior study, as the study investigators [16] acknowledged that their sample size was small (*N* = 18). The significant difference in body weight between the intervention and control groups may have been attributable to the actual intervention components or to frequent contact that participants experienced in the form of daily text messages for skill training and 3–4 text messages weekly for self-monitoring [16]. A possible reason contributing to ineffective interventions in 3 other prior studies may have related to poor attendance in face-to-face group meetings due to transportation barriers, child care issues [14], job schedules, and family illness or death [15]. Cavallo et al., reported that 37% of participants [17] and Krummel et al., reported that 43% of participants [15] attended at least 1 group meeting. Walkers et al., reported that 50% of participants attended 5 to 12 of 13 weekly group meetings and the rest attended mainly 2–3 weekly group meetings [14].

To have a broad public health impact on obesity, we designed *Mothers In Motion (MIM)* that may have potential for adoption, implementation, and sustainability in major community-based programs such as WIC. Recently, *MIM* intervention videos (described later) have been disseminated to WIC nationwide via a shared website. *MIM* aimed to help low-income overweight and obese young mothers prevent further weight gain via promotion of stress management, healthy eating, and physical activity. We chose to deliver video intervention via DVD format because our prior studies consistently showed that 99% of WIC mothers had access to a DVD player and TV at home. Watching intervention videos via DVDs at home could provide flexibility and convenience for intervention participation. We opted not to use social media because we have learned that WIC mothers’ access to the Internet is episodic rather than continual. Also, Facebook might not be an acceptable mode of intervention by the target audience, which is supported by 2 recent studies of low-income young mothers with young children. These studies showed only 11 [18] and 30% [17] intervention participants logged into Facebook. Another component of our *MIM* intervention was peer support group over the phone instead of face-to-face group meetings. This paper presents *MIM* intervention effect on body weight (primary outcome) and summarizes lessons learned.

## Methods

A detailed description of the study setting, inclusion and exclusion criteria, recruitment and randomization has been published [19, 20]. Below, we briefly describe the study procedure, which was approved and monitored by Michigan Department of Health and Human Services and Michigan State University Institutional Review Boards.

### Setting and participants

Our participants were recruited from 7 WIC offices (4 local WIC agencies) in Michigan. These local WIC agencies serve low-income clients (~76% with annual household income at or below 130% of the federal poverty line). Participants were non-pregnant women who were between 6 weeks and 4.5 years postpartum, non-Hispanic Black or White (hereafter referred to as Black or White), 18–39 years old, free of type 1 or 2 diabetes per self-reports, able to walk more than 1 block without resting, and overweight or obese. BMIs were calculated using measured height and weight, between 25.0 and 39.9 kg/m^2^.

### Recruitment and randomization

Recruiters were peers (WIC mothers) who were trained to be culturally sensitive to the target audience [21]. They personally invited women who came to our collaborating WIC offices to be screened. We applied sequential screening (screening I and II) to minimize the potential for high dropout, which commonly occurs in studies with this population. Screening I: our peer recruiters offered consent documents to eligible women only if these women could verbally describe their understanding of the study activities. Screening II. Consented women were asked to complete a baseline phone interview and return to the WIC office where they had been recruited to be randomized within 3 weeks of signing the consent form. Our randomization protocol utilized a variable permuted-block algorithm [22, 23]. Participants were randomized to either an intervention or comparison group (2:1 ratio).

### Intervention

A detailed description of intervention has been published [19]. Below, we briefly describe the intervention.

#### Theory-based culturally sensitive videos

The video featured 4 overweight and obese WIC mothers (3 Blacks, 1 White, hereafter referred to as featured mothers) who met the study criteria. We put their testimonies and demonstrations of making positive lifestyle behaviors into 10 video lessons in a DVD format. Each skill-oriented lesson has a specific topic with minimal redundancy among lessons. Each lesson had 3 components: interactive information (~2 min), vignettes (~17 min), and action planning (~40 s). There were 4 lessons for stress management, 5 lessons for healthy eating, and 1 lesson for physical activity both indoors and outdoors. Nearly 50% of intervention contents included the featured mothers and their young children (aged 3–9) demonstrating positive lifestyle behavioral changes.

#### Peer support group teleconference moderator training

The peer support group teleconference moderators attended 3-day in-person trainings. The motivational interviewing trainer was a WIC administrator with a masters degree in counseling and expertise in motivational interviewing. The training covered both motivational interviewing and group facilitation skills via a mix of didactic and practice activities. After the training, each moderator took part in 4 supervisory sessions to practice leading a mock peer support group teleconference with members of the study’s peer advisory group by phone. After each practice call, the motivational interviewing trainer provided immediate feedback to moderators for improvement. **Fidelity**. Over the course of the intervention, the trainer and moderators listened to the same 25% randomly selected peer support group teleconference recordings/per month using the same fidelity sheet to evaluate adherence to motivational interviewing skills. The trainer also identified strengths and provided specific feedback for areas of improvement. The moderators attended a booster training 4 times/per year: 2 trainings by phone (months 3 and 9) and 2 in person (months 6 and 12).

#### Intervention group

Intervention participants received a 16-week intervention. They were told to stop watching videos or attending peer support group teleconferences if they became pregnant during the trial because our intervention was not designed for pregnant women. **Videos**. Intervention participants watched 1 designated video lesson (via DVD format) weekly (weeks 1–4) or every other week (weeks 5–16) at home. After watching a video lesson (~20 min), they answered 3 questions on a worksheet by circling tips that they planned to apply to their daily life, then mailed the worksheet to the study office using pre-stamped envelopes as evidence of having watched the video lessons. They were also encouraged to set goals and self-monitor progress of making positive behavioral changes. **Peer support group teleconferences** . Each peer support group teleconference had 10 women who remained in the same cohort for the 16-week intervention. Women called into the peer support group teleconferences weekly (weeks 1–4) or every other week (weeks 5–16) to discuss contents (e.g., skills learned) in the designated video lesson that they had watched the prior week. To increase participation, we provided 2 different times (1 in mid-morning or early afternoon and 1 in the evening) for participants to call. Each peer support group teleconference session lasted about 30 min; the sessions were audio recorded with permission from the participants for the purpose of monitoring intervention fidelity. We recorded attendance for the purpose of monitoring intervention participation.

#### Comparison group

The comparison group received printed materials on stress management, healthy eating, and physical activity from standard reliable sources, for example, www.ChooseMyPlate.gov. They also received a 10-min DVD containing information about food and home safety.

### Measurement

Data were collected via phone interview (survey questions including process evaluation) or in person (body weight). This paper focuses on primary outcome (body weight) and process evaluation data.

#### Demographics

Demographic data were collected using a pencil-and-paper survey when women were screened for participation at the WIC office. The demographic variables included birth date of the participants and their youngest child, race, current breastfeeding, number of biological children (used to assess parity), smoking, education, and employment status. To calculate participants’ age and postpartum status (age of the youngest child), we used the date the participant completed the demographic survey minus the self-reported birth date.

#### Height and body weight

Height and body weight were measured to calculate BMI. The principal investigator (Chang) used the NHANES anthropometric manual [24] to train the recruiters (via role-playing) to measure height and weight until we reached inter-rater reliability ≥ 95%. Booster training was conducted every 2–4 weeks in person while Dr. Chang performed site visit to maintain fidelity. **Height** . A wall-mounted stadiometer was used to measure participants’ height without shoes to the nearest 0.1 cm. **Body weight**. We collected body weight data in person at 3 time points: baseline (T1, at screening), immediately after (T2) and 3 months (T3) after the 16-week intervention. Study participants returned to the office where they had been recruited to get their body weight measured (T2 and T3). For those who could not return to the WIC office, we made a home visit to measure their body weight. An electronic digital scale (Seca 869, Germany) was used to measure weight to nearest 0.2 lbs while participants wore light clothing and no shoes.

#### Process evaluation

After completion of T2 phone interview, participants were asked to complete a process evaluation via phone interview. Table 1 presents process evaluation items. **Intervention participants** . ***Reactions to intervention videos***. Participants were asked about their reactions to the intervention videos (4 items; 1 = strongly disagree, 4 = strongly agree). ***Mothers In Motion Program Satisfaction***. We evaluated participants’ satisfaction with *MIM* (*3* items; 1 = strongly disagree, 4 = strongly agree). ***Mothers in Motion program impact***. Participants were asked about overall *MIM* program impact on their personal life (6 items; 1 = strongly disagree, 4 = strongly agree). ***Usefulness of the worksheet***. We used 1 item to ask participants to evaluate the usefulness of the worksheet (1 = strongly disagree, 4 = strongly agree). ***Watching videos with children and partner/husband or sharing any video lessons with relatives or friends***. We asked participants whether they watched each video lesson with children and partner/husband or shared any video lessons with their relatives or friends (10 items, 1 item/per video lesson, yes/no). ***Reasons for any video lesson not helpful***. We used 1 open-ended question to gather such information. ***Reasons for not watching all 10 video lessons***, ***returning all 10 worksheets or dialing in all 10 peer support group teleconferences***. We asked women reasons for not watching all 10 video lessons (2 yes/no items, 1 open-ended question) and reasons for not retuning all 10 worksheets (5 yes/no items, 1 open-ended question) that were used as evidence of having watched intervention video lessons. We also asked reasons for not dialing all 10 peer support group teleconferences (5 yes/no items, 1 open-ended question). ***Contamination***. Finally, we ask “Do any of your relatives or friends who you shared the DVDs with participate in the *Mothers In Motion* program? (yes/no).” **Comparison group**. ***Read printed materials***. For the comparison group, we asked if they read the printed materials (3 items; 1 = none of it, 4 = all of it). *Reasons for not reading all printed materials*. We also asked reasons for not reading all printed materials (3 yes/no items, 1 open-ended question). ***Contamination***. We asked whether they had watched any of our *Mothers in Motion* DVDs (1 item, yes/no).

**Table 1:** Results of Process Evaluation with Study Participants

Intervention Groups		
Reactions to intervention videos (*n* = 213)	Strongly disagree/disagree (%)	Agree/strongly agree (%)
I trusted information in the videos	0	100
I related to the moms in the videos	5.1	94.9
The stories in the videos were believable	0.9	99.1
The stories in the videos were inspiring	3.8	96.2
*Mothers In Motion* program satisfaction (*n* = 213)		
I have been very satisfied with what I learned in the program	2.3	97.7
I have been very satisfied with what I experienced in the program	2.8	97.2
I have learned a great deal from the program	6.0	94
*Mothers In Motion* Program impact (*n* = 213)		
My life has changed for the better	8.9	91.1
I have a happier and healthier family	12.2	87.8
My children have been eating more healthy foods	12.7	87.3
My children have been eating less junk foods	15.0	85.0
I have been doing more physical activity with my children	9.0	91.0
I have noticed my children being more active	14.5	85.5
Usefulness of the worksheets (*n* = 213)		
The worksheets were useful to me	6.1	93.9%
Watching videos with children and partner/husband or share videos with relatives or friends (*n* = 213)	No %	Yes %
Children	10 lessons ranged from 78.0 to 86.5	10 lessons ranged from 13.5 to 22.0
Partner/husband	10 lessons ranged from 86.3 to 91.5	10 lessons ranged from 8.5 to 13.7
Relatives or friends	10 lessons ranged from 68.4 to 78.1	10 lessons ranged from 21.9 to 31.6
Reasons for any video lessons not helpful^a^ (*n* = 153 who reported 1 or more video lessons not helpful)	Already knew the tips or information, already applied tips in the videos before joining the program, could not apply tips to young baby	
Reasons for not watching all 10 video lessons (*n* = 33 who did not watch all 10 video lessons)	No %	Yes %
Not interested in the topic	87.9	12.1
Could not find the DVDs	87.9	12.1
Other reason^a^	Illness or death of a family member, custody, working too much, busy	
Reasons for not returning all 10 worksheets (*n* = 58 who did not return all 10 worksheets)	No %	Yes %
Forgot	37.9	62.1
Filling out the worksheet was too much of a bother	94.8	5.2
No mail box near my apartment or house	91.4	8.6
Cannot find the worksheets	81.0	19.0
Never received the worksheets that were supposed to be in the *Mothers In Motion* Binder	96.6	3.4
Other reason^a^	Illness or death of a family member, family issues, moving, busy	
Reasons for not dialing in all 10 peer support group teleconferences (*n* = 179 who did not dial in all 10 peer support group teleconferences)	No %	Yes %
Forgot	66.1	33.9
Too busy	23.2	76.8
Time conflict	24.2	75.7
Not enough cell phone minutes	89.8	10.2
Not access to a phone	85.9	14.1
Other reasons^a^	Illness of a family member, family issues, personal problems (e.g., anxiety, depression)	
Comparison Group		
Read printed materials about … (*n* = 98)	None of it/some of it	Most of it/all of it
Stress management	34.2	65.8
Healthy eating	32.2	67.9
Physical activity	42.0	58.0
Reasons for not reading all stress management, healthy eating and physical activity printed materials (*n* = 98)	No %	Yes %
Not interested in the topic	79.6	20.4
Could not find the printed materials	90.8	9.2
Did not like reading any printed materials	85.4	14.3
Other reasons^a^	Busy	

^a^open-ended question

### Statistical analysis

Based on our pilot *MIM* [25], we proposed to detect a minimum effect of 2.8 lbs difference between the intervention and comparison groups. This provided an effect size of 0.26 and resulted in a total of 291 (intervention group = 193, comparison group = 94) to achieve power at 0.80, with type I error < 0.05 (2-tailed). During the trial, we experienced a higher dropout rate than expected. To ensure adequate sample size (*N* = 291) for the final data analysis, we enrolled a total of 612 women. Our data analysis included 569 women (intervention: 387, comparison: 182) because our plan called for exclusion of data from the 43 women who self-ported becoming pregnant during the trial: intervention = 23 (23/410 = 5.6%), comparison = 20 (20/202 = 9.9%). NCSS software (version 11) was used for data analysis. We performed *t*-test for continuous variables and Chi-squared test for categorical variables on demographic data. Also, we applied descriptive analysis to analyze process evaluation data. To assess both between and within intervention effects on body weight, we performed general linear mixed model for repeated measures, which is a partial intention-to-treat analysis (ITT), using baseline measures as adjusting covariates. True intention-to-treat analysis using imputation approaches (such as last observation carried forward) can reduce the power of the analysis as the number of missing values increases. Therefore, we performed general linear mixed model analysis for repeated measures without any ad hoc imputation as our approach to ITT. Simulation studies have indicated that equal or more power occurs using mixed models with no imputation (partial ITT: use all available data) than using imputation methods [26]. Finally, we performed content analysis to identify common themes from open-ended questions.

## Results

### Demographics

**Fig. 1:**
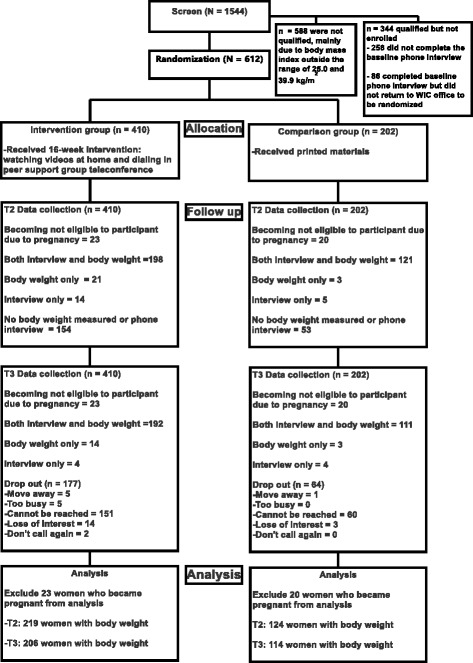
Consort Chart

Table 2 presents demographic characteristics of the study participants (*N* = 569). There were no significant differences between the intervention and comparison groups. At baseline, the mean age of the participants was 28.5 ± 5.0 years (intervention: 28.4 ± 5.0, comparison: 28.9 ± 5.0). The mean parity was 2.3 ± 1.3 (intervention: 2.3 ± 1.2, comparison: 2.2 ± 1.3). Figure 1 presents a CONSORT chart of the study.

**Table 2:** Study Participant Characteristics (*N* = 569)

Characteristics	Intervention (n = 387)		Comparison (n = 182)		*P* value	Total	
Demographics	Mean	SD	Mean	SD		Mean	SD
Age (years)	28.38	5.02	28.86	5.04	0.29	28.53	5.03
Postpartum status (age of the youngest child, in years)	1.64	1.24	1.88	1.33	0.07	1.71	1.27
Body mass index (BMI, Kg/m^2^)	32.16	4.35	31.74	4.15	0.27	32.03	4.29
	n	%	n	%		n	%
Body mass index category					0.71		
Overweight (BMI 25.0-29.9)	137	35%	64	35%		201	35%
Obese category 1 (BMI 30.0-34.9)	139	36%	71	39%		210	37%
Obese category 2 (BMI 35.0-39.9)	111	29%	47	26%		158	28%
Race					0.77		
Blacks (non Hispanic)	306	79%	142	78%		448	79%
Whites (non Hispanic)	81	21%	40	22%		121	21%
Current breastfeeding					0.98		
Yes	64	17%	30	16%		94	17%
No	323	83%	152	84%		475	83%
Parity					0.62		
1	128	33%	64	35%		192	34%
2 or more	259	67%	118	65%		377	66%
Smoking status					0.20		
Never smoked	167	43%	93	51%		260	46%
Smoked, but quit	114	30%	47	26%		161	28%
Smoker	106	27%	42	23%		148	26%
Education					0.11		
8th grade or less	0	0%	1	1%		1	1%
Some high School	51	13%	14	8%		65	11%
High school graduate	75	19%	46	25%		121	21%
Some college or technical school	186	48%	84	46%		270	47%
College graduate or higher	75	19%	37	20%		112	20%
Employment status					0.46		
Full time	81	21%	44	24%		125	22%
Part time	81	21%	40	22%		121	21%
Unemployed	76	20%	42	23%		118	21%
Homemaker	110	28%	38	21%		148	26%
Self-employed	9	2%	7	4%		16	3%
Student	23	6%	8	4%		31	5%
Other	7	2%	3	2%		10	2%

### Cohort retention and intervention participation

The overall cohort retention rate at the final data collection was 56.2% (320/569, intervention: 53.2% [206/387], comparison = 62.6% [114/182]). **Intervention participation**. We used return-mail worksheets as evidence of watching videos. The average was 5.5 (SD = 4.5) of 10 worksheets; 73.4% (284/387) women returned at least 1 worksheet. Of women who returned the worksheets, 15.2% returned 5–9 worksheets and 62.4% returned 10 worksheets. We recorded attendance in the peer support group teleconferences. The average was 2.6 (SD = 3.4) of 10 calls; 52.9% called in at least 1 time. Of women who dialed in the peer support group teleconference at least once, 35.0% dialed in 5–9 times and 12.4% dialed in 10 times. Table 1 presents results of incomplete intervention participation (not watching all 10 video lessons, not returning all 10 worksheets, or dialing in all 10 peer support group teleconferences).

### Weight differences

At recruitment, 35.3% of participants were overweight and 64.7% were obese. The mean weight was 190.2 ± 1.4 lbs (intervention: 191.8 ± 30.0, comparison: 188.5 ± 29.1); the mean body mass index (BMI) was 32.2 ± 4.4 (intervention: 32.2 ± 4.4, comparison 31.7 ± 4.2; 64.7% of the participants were obese). Table 3 presents weight differences between the intervention and comparison groups adjusting for baseline body weight. At T2 (immediately after the 16-week intervention), no significant weight differences were found between the intervention and comparison groups when controlling for baseline body weight. Similarly, at 3 months after the 16-week intervention, no significant weight differences were found between the intervention and comparison groups when controlling for baseline body weight.

**Table 3:** Body Weight (Mean and SD) Adjusted for Baseline Levels

Baseline (intervention = 382, comparison = 182)				
	Intervention M (SD)	Comparison M (SD)	Effect Size^a^	95% Confidence Interval
BMI	32.17 (4.35)	31.74 (4.15)	NA	NA
Body weight (lbs)	191.83 (30.04)	188.48 (29.05)	NA	NA
Immediately after the 16-week intervention (intervention = 219, comparison = 124)				
BMI	31.66 (1.83)	31.66 (1.84)	0.00	−0.22, 0.22
Body weight (lbs)	188.48 (10.84)	188.52 (10.87)	0.00	−0.22, 0.22
3 months after the 16-week intervention (intervention = 206, comparison = 114)				
BMI	31.62 (1.80)	31.53 (1.79)	0.05	−0.18, 0.28
Body weight (lbs)	188.25 (10.64)	187.74 (10.59)	0.05	−0.18, 0.28

^a^Bias corrected (Hedges)

### Process evaluation

Of participants, 213 (213/387 = 55.0%) intervention participants and 98 (98/182 = 53.8%) comparison participants completed the process evaluation. Table 1 presents results of process evaluation. Although sharing intervention videos was specifically disallowed during the study period, 8% of intervention participants admitted sharing videos with comparison participants and 6% of comparison participants reported having watched the intervention videos at T2 process evaluation.

## Discussion

Up to date, *MIM* is one of the first studies with adequate power to test effectiveness of an intervention in low-income overweight and obese young mothers with young children and in a real-world situation. Our participation rate for video watching might have been underestimated because we observed that many women who dialed in the peer support group teleconference were able to describe the contents in the videos but had not returned the worksheet. Nevertheless, our participation rate was higher than previous intervention studies that utilized Facebook (11–30%) [17, 18] and in person group meetings [14, 15, 17], suggesting the importance of intervention delivery that is flexible and convenient for the target audience. Our participation rate for the peer support group teleconference did not meet our expectations, in spite of careful planning and adjustments to participant needs. Our prior work (unpublished data) and other study of focus group discussion with low-income overweight and obese young mothers with young children consistently showed that these women preferred a group intervention with inter-participant support [15]. Also, a recent integrative review article showed that low-income obese women voiced a preference for group interventions led by peers or medical professionals [27]. We provided group sessions via phone to minimize barriers to participation (e.g., lack of transportation and child care). We also provided 2 different times to dial in based on our pilot *MIM* process evaluation. Unfortunately, we experienced very minimum improvement of adherence when comparing to our pilot *MIM* for the same reasons: too busy and time conflict. Our group teleconference was scheduled based on the moderator’s availability because it was not feasible to schedule a group teleconference to fit in each participant’s schedule. Our low participation rate of group intervention is consistent with findings of prior studies of low-income young mothers with young children [14, 15, 17], suggesting that group intervention delivered either in person or via phone is not feasible for this population.

### Possible reasons for no intervention effect

Consistent with results of most prior studies of low-income overweight and obese young mothers [14, 15, 17], we did not find a significant difference in body weight between the intervention and comparison groups.

### Intervention-related reasons

As part of monitoring fidelity of using motivational interviewing, we listened to a random selected peer support group teleconference recordings (25%) and identified common themes. We also randomly selected 25 intervention participants who completed T3 to further evaluate the intervention (semi-structure phone interview and using qualitative methods to identify common themes, unpublished data). Based on listening to recordings and results of the 25 interviews, we have identified possible reasons contributing to less favorable intervention effects.

#### Challenges in setting goals and choosing action plans

In our pilot *MIM*, we collected worksheets that asked participants to write down their goals, action plans and self-monitor progress of stress, dietary intake, or physical activity daily (by circling 1 of the 3 options: not so great, so so, great, unpublished data). We learned that most participants did not know how to set goals. For example, participants would write down ‘finding a job’ as a goal of reducing stress. Thus, for the present study, we did not collect the worksheets with self-monitoring of goals because we were concerned about adding additional burden for participants and some women with low literacy or learning disability might be frustrated by this task, thus decreasing their interest in making positive changes. Instead, we asked women to circle tips listed on a worksheet that they would like to apply in their daily life. We provided example goals for stress management, healthy eating and physical activity on the printed materials. In each video lesson, we provided additional examples of goal setting and action plans to achieve goals. We strongly encouraged intervention participants to set goals and develop action plans on a weekly basis to better manage stress, eat healthier, and be more physically active. Still, some women were not sure how to set goals for healthy eating and physical activity. Even when they did set goals, they had difficulty identifying tips learned from video lessons that they could apply to achieve their goals. Future studies may consider providing a list of goals that are commonly set by the target audience with specific and practical action plans from intervention contents so that participants can readily identifying the strategies that would help them reach their personal goals. A possible option to help women self-monitor progress of achieving their goals is to use text messages instead of paper. However, this would only apply to populations with reliable Internet connections and continuous phone service.

#### Not addressing specific psychosocial needs of the target audience

Of the study sample, 58.4% (333/569: intervention = 58.9 [228/387], comparison = 57.7% [105/182]) had clinical depression per CESD depression scale data (data not shown). Depressive symptoms are strongly associated with everyday stressors (e.g., parenting worries, interpersonal conflict) [28, 29] and negative thinking [30–32] in low-income young mothers with young children. However, our intervention did not address tips for overcoming depressive symptoms, which is associated with increased appetite [33, 34], less healthy eating and emotional eating [35–38] and obesity [39]. We observed that many women had overwhelmingly negative thoughts and continued to express high stress and depressive moods throughout the intervention, thus, they had minimal interest in discussing healthy eating or physical activity. Our stress management topics covered tips for dealing with daily hassles (e.g., handling piles of laundry and not being a “perfect mom”), time management (e.g., set a priority and make a to-do-list), and parenting tips. Prior studies of overweight and obese young mothers regardless of income level that included similar topics of stress management showed no intervention effect on body weight. Results of our and prior studies suggest that specific needs for low-income overweight and obese young mothers may have been overlooked, such as addressing negative thoughts in relationship to stress and depression.

#### Intervention covering making changes with young children

We had about 50% intervention contents focusing on healthy eating and physical activity in mothers and their young children (aged 3–9) based on our pilot *MIM* intervention evaluation [25]. Our pilot intervention participants said that children are a very important part of their life, thus it is critically important to have intervention contents covering making positive changes for both mothers and children (family). The significant focus on family may have had negative impact on our intervention. In fact, many women in our teleconferences were interested in talking about helping their children eat healthier and be more physically active, but not themselves. A prior study of non low-income overweight and obese young mothers conducted family intervention plus motivational interviewing and resulted in no significant difference in mothers’ body weight between the intervention and comparison groups [40].

#### Not seeing immediate benefits

Our intervention emphasized making small and gradual positive lifestyle behavioral changes. As we observed that during the peer support group teleconferences, many women expressed disappointment or frustration of making positive lifestyle behavioral changes and wanted to give up because they were not aware the benefits of making a small change (e.g., having more energy from eating healthier). They were willing to continue when moderator of peer support group teleconference identified the benefits of change that they had experienced. Some participants might have joined the program for the purpose of weight loss. They might have failed in the past and were looking for quick fix. When they did not see the anticipated results of weight loss or experienced plateaus, they might have simply given up.

#### Relapse

Our intervention did not cover tips for preventing and recognizing relapse. Many women experienced relapse and were not aware of it as they shared with us that our intervention was helpful at the beginning but did not work after a while.

#### Allowing participants to make up watching intervention videos

Allowing women to make up video watching after the 16-week intervention might have maximized participation and minimized drop out but might have had a negative impact on the intervention’s effect on body weight. Our target audience is very mobile and lives stressful daily lives. At the start of the study, some of our participants thought if they fell behind the intervention schedule, they would be automatically dropped from this study. Thus, we allowed them to continue watching the intervention videos after the 16-week intervention. This approach mimics the real-world situation, where women would have an opportunity to make up missed intervention components.

### Possible reasons unrelated to intervention

#### Interval between expressing interest to participation and beginning study activities

The interval between expressing interest to participation and beginning study activities ranged from 2 to 6 weeks because we had to group 10 women into a group for the purpose of peer support group teleconferences. It is possible that some intervention participants might have lost interest in engaging in intervention activities due to the delay.

#### Underreporting becoming pregnant

Our intervention participants were about 2 times less likely to report becoming pregnant during the trial than the comparison participants. This suggests that our final analysis might have included body weight of women who had become pregnant during the trial, especially for intervention group.

#### Contamination

Our intervention participants admitted sharing the intervention videos with comparison participants during the process evaluation at T2 data collection. Unfortunately, we did not collect contamination data at T3. Many intervention participants told our peer recruiters reasons for sharing the *MIM* videos with others because they found these videos to be relatable, relevant, practical and helpful to manage stress, eat healthier and be more physically active with their young children. Thus, they felt that it was very important to share *MIM* intervention videos with others. Intervention contamination is an inherent challenge for studies conducted in real-world situations, especially when intervention participants find intervention materials to be valuable. When designing the present study we thought about utilizing a cluster randomization to minimize potential contamination. However, this approach was not feasible for several reasons. First, the demographic characteristics of WIC clients were different across agencies where we sampled study participants. For example, some WIC offices serve mostly rural clients while others serve clients in urban areas; some serve predominantly Whites but others serve predominately Blacks. Also, the size of collaborating WIC agencies were different. One agency has only 1 WIC office, others have 2–5 WIC offices.

#### Recruitment messages

Our recruitment emphasized stress management and having a happier and healthier family and deemphasized healthy eating and physical activity because we experienced significant challenges in recruitment when we emphasized healthier eating and physical activity. Thus, it is possible that many women participated in the study for the purpose of stress management and feeling happier to deal with a life crisis, e.g., relocation, going through divorce, relationship change with boyfriend, fighting custody, taking care of ill family members, or becoming ill themselves. These challenge life events might have prevented them from continuing to engage in the study activities.

#### Taking depression medication

More than 50% of our study sample had clinical depression (described earlier). However, we did not ask our participants if they were taking any depressive medications, which often increase weight gain [41, 42]. Thus, our intervention effect might have been affected by depression medications.

##### **Reasons for not applying learned tips**

How to motivate the target audience applying tips learned from the videos to daily life was a challenge. We learned that some women were hesitant to make changes because they were afraid of trying something new or failing. Others thought that they already knew and practiced the tips learned from the videos prior to joining *MIM*, ate healthy, and were physically active; thus, they were unwilling to make new changes.

### Limitation

*MIM* was funded as a translational research project. To maximize external validity, we recruited women between 6 weeks and 4.5 years postpartum. These women may have different needs between early and late postpartum and our intervention was not designed to meet the specific needs of either early or late postpartum women. Our participants were recruited from narrow geographic locations, which limited generalizibility of the study findings. Also, due to the recruitment emphasis on stress management and a happier and healthier family, we might have recruited women with higher stress and depression than other WIC mothers who did not participate in the study.

#### Recommendations for future studies

Based on ours and previous studies, future studies of low-income overweight and obese young mothers may seek alternatives to group intervention modalities, either in person or over the phone. For example, one-on-one intervention over the phone with flexible scheduling or newer technologies for interpersonal contact may better fit the needs of this target audience. Future studies may consider covering topics related to substituting positive thoughts for negative thoughts to help low-income overweight and obese women effectively manage feelings (stress and depressive symptoms) that affect healthy eating and physical activity. Also, researchers may specify the immediate benefits of making change that are tailored to their target audience and ask women to consider those benefits on a regular basis to help them identify positive changes being made, thus motivating them to continue making changes. Also, individual coaching over the phone or in person may be used to facilitate goal setting and action planning to achieve goals and identifying individual motivation to adhere to the goal. Finally, future studies may ask potential participants to view a short video with their peers’ testimonies to motivate them to overcome barriers to making changes (e.g., “I already know everything”). We have observed that our target population are more likely to make positive lifestyle behavioral changes if messages are from their peers rather than professionals because they feel that if their peers can do it, they can do it too.

## Conclusion

Lifestyle behavioral intervention studies that aim to help low-income young mothers with young children manage weight are a top priority for public health due to the disproportionally high prevalence of overweight and obesity in this population. However, many challenges (e.g., intervention contamination) present challenges to rigorous evaluation of intervention effects.

## Abbreviation

BMI: Body Mass Index
ITT: Intention to Treat Analysis
MIM: *Mothers In Motion*
WIC: The Special Supplemental Nutrition Program for Women, Infants, and Children
